# Flood-Related Multimedia Benchmark Evaluation: Challenges, Results and a Novel GNN Approach

**DOI:** 10.3390/s23073767

**Published:** 2023-04-06

**Authors:** Thomas Papadimos, Stelios Andreadis, Ilias Gialampoukidis, Stefanos Vrochidis, Ioannis Kompatsiaris

**Affiliations:** Information Technologies Institute, Centre for Research and Technology Hellas, 6th km Charilaou-Thermi Rd, 570 01 Thessaloniki, Greece

**Keywords:** multimodal fusion, graph neural networks, social media, benchmark dataset, crisis management

## Abstract

This paper discusses the importance of detecting breaking events in real time to help emergency response workers, and how social media can be used to process large amounts of data quickly. Most event detection techniques have focused on either images or text, but combining the two can improve performance. The authors present lessons learned from the Flood-related multimedia task in MediaEval2020, provide a dataset for reproducibility, and propose a new multimodal fusion method that uses Graph Neural Networks to combine image, text, and time information. Their method outperforms state-of-the-art approaches and can handle low-sample labelled data.

## 1. Introduction

**Figure 1:**
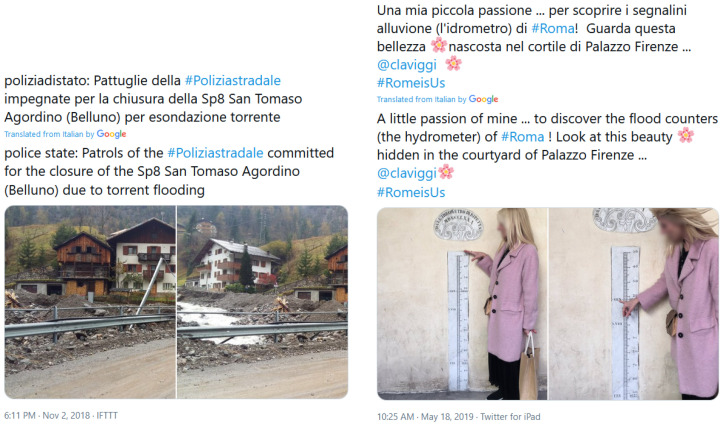
Example of tweets that are relevant (**left**) or not relevant (**right**) to a flood event.

Social media has recently become a digital lifeline used to relay information and locate survivors in crucial situations [1,2,3]. Billions of images and texts that capture a wide range of events, such as floods (left example in Figure 1), are constantly uploaded to social media platforms. However, the large streams of published posts carry lots of noise because of the metaphorical use of incident-related words (e.g., right example in Figure 1), making it hard to collect high-quality information. To tackle this problem, automatic estimation of a post’s relevance can contribute by separating relevant posts from irrelevant and thus filter out unrelated information [4] to the crisis event.

Handling a large volume of social media data during a crisis can be a challenging task for the first responders. With limited capacity to manage all calls and prioritize response, real-time collection of relevant social media information becomes crucial as time is of the essence. Furthermore, identifying damage and human casualties is essential to efficiently allocate resources and save as many lives as possible. For example, during Hurricane Sandy in 2012, people turned to social media to communicate when traditional information sources were limited or unavailable [5]. Similarly, during Hurricane Harvey in 2017, a woman was rescued after tweeting for help when 911 was not responding [6]. During the 2019–2020 Australian bushfires and the 2020 Beirut explosion, social media were instrumental in sharing updates, requesting assistance, and organizing rescue operations and relief efforts [7,8]. Despite the potential benefits of social media, filtering relevant information from the overwhelming volume of data remains a significant challenge for first responders during a disaster. Manual classification of useful information is not feasible, as it would divert valuable resources away from more critical tasks.

Since social media posts constitute multimedia data and contain multiple types of information, such as text, image, and other metadata, we have proposed a task at the MediaEval Multimedia Evaluation benchmark 2020 (https://multimediaeval.github.io/editions/2020/ (accessed on 1 April 2023)), namely the Flood-related Multimedia Task (https://multimediaeval.github.io/editions/2020/tasks/floodmultimedia/ (accessed on 1 April 2023)), which encourages participants to use multiple modalities, such as textual and visual ones, with a geographical restriction in Italy, in a language that goes beyond the traditional use of English. Additionally, we propose an alternative relevance estimation approach that fuses multimodal inputs with Graph Neural Networks. Previous state-of-the-art semi-supervised learning techniques were used, to jointly train neural networks using limited labelled and unlabelled data with graph augmentation, which can be trained with stochastic gradient descent and efficiently scaled to large graphs. The proposed work has a regularisation term for generic neural network architectures that enforces similarity between nodes in the graphs [9].

In this paper we:

- Present the creation and public release of a flood-related multimedia benchmark dataset, which is in Italian (rather than typically in English) and is annotated by experts
- Describe the Flood-related Multimedia Task 2020 and comment on the challenges faced by the participants
- Propose a novel Graph Neural Network approach for relevance estimation and evaluate it against state-of-the-art methods to show its outperformance

The organisation of the paper is given as follows. Section 2 presents a review of relevant datasets and the latest relevance estimation models in crisis events. Section 3 describes the methodology of the proposed work and Section 4 continues with the presentation of the Flood-Related Multimedia Task and the respective dataset. Section 5 provides the main baselines that our proposed model is compared to, the settings of the methodology and the results of the evaluation, while Section 6 concludes the paper.

## 2. Related Work

### 2.1. Related Dataset & Competitions

The big interest of the scientific community in utilising online multimedia for disaster management has led to relevant benchmark evaluation tasks and challenges for researchers. The newly introduced “Disaster Scene Description and Indexing” (DSDI) task [10] of the TREC Video Retrieval Evaluation (TRECVID) 2020 (https://www-nlpir.nist.gov/projects/tv2020/ (accessed on 1 April 2023)) provided participants with circa 5 h of video of real-world disaster events and asked them to return a ranked list of the top video clips that include a natural disaster-related feature from a predefined set. ImageCLEF 2017 [11] proposed the ImageCLEFremote pilot task (https://www.imageclef.org/2017/remote (accessed on 1 April 2023)), whose objective was to explore Sentinel Copernicus satellite images for predicting the human population, which is also applied in planning disaster responses. Contrary to these two challenges, our proposed task focused on social media data, which have been proven key communication channels during crises.

Considering social media data, Kaggle initiated on 20 December 2019 an open competition (https://www.kaggle.com/c/nlp-getting-started/overview (accessed on 1 April 2023)) about natural language processing with disaster tweets. Given a dataset of 10,000 hand-classified tweets, the participants are challenged to build a machine learning model that predicts which tweets are about real disasters and which ones are not. Furthermore, the MediaEval Multimedia Evaluation benchmark (https://multimediaeval.github.io/editions/2020/ (accessed on 1 April 2023)) offers, since 2010, tasks that are related to multimedia retrieval, analysis, and exploration and in 2017 introduced the “Multimedia Satellite Task”, which lasted for three years, focused on the natural disaster of floods and included subtasks that concerned social media data as well, apart from satellite images. The goal of the “Disaster Image Retrieval from Social Media” (DIRSM) subtask [12] was to design an algorithm that can identify which images, obtained from social media streams (Flickr, Twitter, Wikipedia), are related to a flooding event. In 2018, the goal of the “Flood classification for social multimedia” subtask [13] was to retrieve all images from social media that provide direct evidence for passability of roads by conventional means, while in 2019 the “Multimodal Flood Level Estimation from News” subtask [14] asked participants to build a binary classifier that predicts whether or not an image contains at least one person standing in water above the knee. In 2020, the “Multimedia Satellite Task” evolved into the task presented in this paper, namely the “Flood-related Multimedia Task” [15]. In contrast to the previous years, the task aimed attention at data from Twitter and invited researchers to use both textual and visual information to classify posts as relevant or not to floods in a specific region. Moreover, the main differences to the above-mentioned Kaggle competition was the addition of images and the selection of exclusively Italian tweets, to encourage data scientists to move away from a focus on English.

Regardless of evaluation tasks and contests, there are also several resources available, with annotated social media data, which can help researchers and technologists to advance research on crisis informatics topics. The resource of [16] consists of Twitter data collected during 19 natural and human-induced disasters and are labelled with the type of information they carry (e.g., reports of casualties, reports of urgent needs, emotional support, etc.). Similar annotation can be found for tweets collected during the 2012 Hurricane Sandy and the 2011 Joplin tornado in [17,18]. A multimodal dataset provided by [19] comprises tweets and images collected during seven major natural disasters and the annotation is extended with labels of damage severity. Human annotation of damage severity levels is also offered in [20], on images collected from Twitter during the 2014 typhoon Ruby, the 2015 Nepal earthquake, the 2016 Ecuador earthquake, and the 2016 Hurricane Matthew. In [21] circa 14,000 tweets posted during hurricanes, earthquakes, floods, and forest fires are labelled with regards to eyewitnesses (e.g., direct/indirect). An alternative resource consists of tweets collected from the 2015 Nepal earthquake and the 2013 Queensland floods, which are human-annotated as relevant and non-relevant [22]. Finally, [23,24] developed consolidated crisis benchmark datasets that include non-overlapping data from several data sources. Unlike the majority of available resources, our proposed dataset does not aim the categorisation of tweets to different types of information, but offers relevant/non-relevant labels, assigned by expert annotators, which can be used to develop models that filter out non-valuable posts and thus improve the quality of crowdsourced information. In addition, the dataset focuses on flood-related tweets in Italian, to overcome the common limitation of training data being in the English language; as seen above, near all available textual datasets are in English [12,17,18,19,21,22], while 16 out of 19 datasets in [16] and circa 95% of tweets in [24] are also in English.

### 2.2. Related Methodologies

Previous studies have predominantly focused on analyzing textual data alone [25,26]. However, in recent years, the field has witnessed an increase in the application of deep multimodal learning, which involves the integration of complementary information from multiple modalities.

For instance, ref. [25] proposed a technique that utilizes Convolutional Neural Networks (CNNs) to identify informative tweets during disasters. They employed word embeddings to extract features from the text, which were then processed using Convolutional Neural Layers and a hidden layer. In contrast, our experimental analysis for text feature extraction employs a combination of CNN-BiLSTM approaches.

Similarly, ref. [27] fused textual and hydrological features to classify tweets as relevant or irrelevant to floods. They used fastText word embeddings [28] and the supervised MUSE method to create multilingual word embeddings [29]. Conversely, we fuse textual data with image and temporal features.

The authors in [30] proposed a multimodal deep learning framework to detect damage-related information in social media posts using text, images, and videos. They utilized an Inception CNN model pre-trained on ImageNet to process images and a pre-trained word embedding model for text processing, which was then fed to a single-layer CNN. In our experiments, we use the VGG19 network to process images and CNN-BiLSTM for text, and fuse them with rich features extracted by BERT [31].

Additionally, ref. [32] introduced a decision fusion technique that combined probability scores of individual text and image classification predictions to arrive at a composite multimodal classification. The text input was processed using BiLSTM, and image predictions were obtained using Inception-v3. After obtaining the image and text representations using the mentioned approaches, the authors concatenated them with Logistic Regression Decision [33]. In contrast, we use graph-based techniques to fuse different modalities.

Another work, ref. [34], utilized two parallel deep learning architectures to learn a joint representation from both text and image modalities. The authors used VGG16 network architecture to extract high-level features from an input image using the fully-connected layer of the network, while a CNN with five hidden layers and different filters was defined for text processing. Two feature vectors obtained from both modalities were then fed into a shared representation followed by a dense layer, before performing a prediction using SoftMax. Instead of concatenating, we extract features from text using BERT and CNN with BiLSTM, fuse them, and then perform a prediction.

The authors of [35] analyzed text, images, and videos from a multitude of social media platforms and applied different machine learning algorithms for floods assessment, to conclude that Random Forest achieved the highest accuracy and in particular for the YouTube videos.

The work in [36] proposed a deep learning-based methodology that aims to assess the flood severity by using text and image data extracted from the social media posts, while the authors of [37] experimented on a Fully Convolutional Network in order to build an event detection system trained on word count time series and coupled with an automated lexicon building algorithm.

Moreover, ref. [38] proposed a flood prediction framework that utilizes both tweets and images from social media. They used a deep learning model to extract features from text and images, and then used these features to predict the likelihood of a flood.

In [39] the authors combined social media images and short text information of crisis-related tweets using the BERT method for text inputs and DenseNet [40] for image inputs. Their proposed Cross-Attention module avoided transferring negative knowledge between modalities, and used stochastic shared embeddings to mitigate overfitting in small data as well as dealing with missing data in some modalities. On the other hand, we use Neural Structure learning to address missing data and overfitting in small data.

Finally, ref. [41] presented a comprehensive overview of the current research on utilizing social media for crisis management. It also included a detailed compilation of case studies where social media, particularly Twitter, served as a crucial source of information for rescuing individuals during flood events.

In contrast, the works that focus on flood prediction from tweets, as I mentioned earlier, are primarily focused on the analysis of textual data from social media to predict the occurrence of floods. While your work may also utilize textual data from social media, it seems to be exploring a more comprehensive approach to disaster response by integrating multiple modalities of information to enhance the accuracy and timeliness of predictions. Additionally, we use a different approach to combine features from multiple modalities with Graph Neural Networks (GNN) [42], as also Table 1 presents. We train neural networks by leveraging structured signals in addition to feature inputs. Structured signals in graphs are commonly used to represent relations of similarity among samples. Graph Neural Networks are a different approach of traditional deep learning, risen in recent years, that operate on graph domain and have the ability to extract multi-scale localised spatial features and compose them to construct highly expressive representations. We present an approach which uses state-of-the-art semi-supervised methods to generate rich feature representations and fuse them with Graph Neural Networks instead of traditional early fusion.

## 3. Methodology

**Figure 2:**
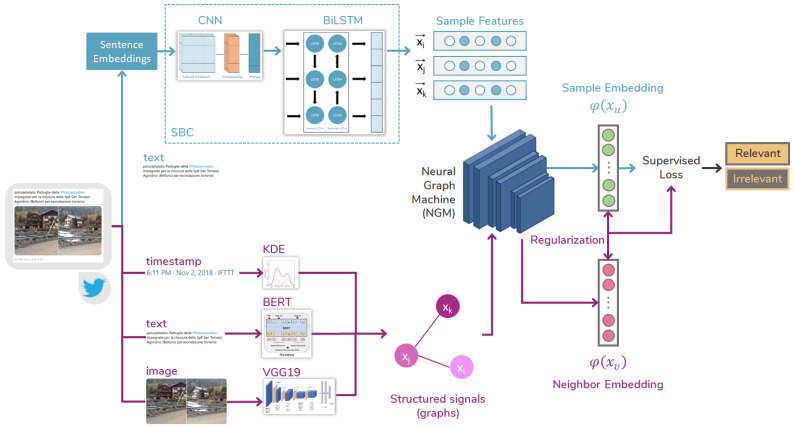
Model architecture.

In this study, we present a classification architecture (see Figure 2) for determining whether a tweet is relevant to flooding events. The architecture takes in three fundamental attributes of a tweet, namely text, image, and timestamp, and outputs a binary prediction of relevance. To extract features from each input modality, we utilize four different neural networks that work in parallel, and transform these features into separate representation vectors. We then employ a Neural Graph Machine (NGM) [9] to combine these high-level representations and apply them to a Sequence-Based Classification model (SBC) [44]. Our approach leverages the ability of NGM to improve label propagation by biasing the network to learn similar hidden representations for neighboring nodes on a graph. Additionally, we propose a novel approach that incorporates the temporal feature in a multimodal fusion classification for social media streams.

Our methodology consists of three parts. In the first part, we extract features from each modality using four different methods: two for text (BERT [31], Word2vec [45], CNN, LSTM), one for images (VGG19 [46]), and one for the temporal feature, which we extract from the publication time of tweets. In the second part, we use the SBC model to process the textual features as sequences and estimate relevance based on text-only inputs. In the third part, we introduce the NGM to fuse the multimodal features and serve as a knowledge base for the SBC to improve regularisation. We elaborate on each of these parts in the following subsections.

### 3.1. Feature Extraction

The proposed model utilizes a combination of two feature extraction techniques—Word Embedding and Bidirectional Encoder Representation from Transformers (BERT)—along with Neural Graph Machine, which is explained in Section 3.3. Word Embedding is a technique that maps words from the vocabulary to real-numbered vectors based on their probability distribution in the corpus. On the other hand, BERT uses an attention mechanism to learn the contextual relations between words in a text. It reads the entire sequence of words at once and is considered non-directional, allowing the model to learn the context of a word based on its surroundings.

In this work, the Word2Vec model is employed to generate a sequence-based vector space, typically of several hundred dimensions, which is then processed by the Sequence-Based Classification model discussed in the next section. For each text $t_{i}$, both Word Embedding and BERT techniques are used to extract features, resulting in a comprehensive representation of the text. The old-fashioned Bag-of-Words approach with term frequency-inverse document frequency (TF-IDF [47]) is not used in this model.

(1) $$T_{i}=BERT\left(t_{i}\right)$$

where $T_{i}\in R^{H}$ is the global max pooling mode for feature extraction.

As far as it concerns the visual modality, since the tweets of the dataset contain an image too, we extract visual concepts from this subset of tweets using Convolutional Neural Networks (CNNs). After internal experimentation, VGG19 [46] was proved to perform better than ResNet152v2 [48], InceptionResNetV2 [49], and Xception [50], thus VGG19 was preferred to be integrated into our model and therefore for each image $m_{i}$ we have:

(2) $$I_{i}=VGG\left(m_{i}\right)$$

where $I_{i}\in R^{D}$ is the global max pooling mode for feature extraction.

**Figure 3:**
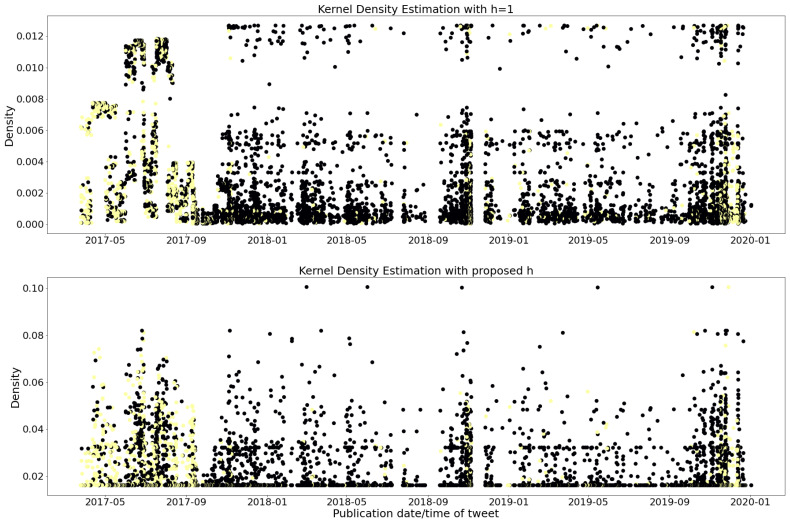
Kernel Density estimation on the MediaEval dataset. Estimation with bandwidth h=1 on the first plot and Sheather and Jones on the second. The horizontal axis shows the time when tweets were posted and the vertical axis the density estimation. Yellow dots represents relevant tweets, while black the non-relevant tweets.

Another feature that seems to be valuable is the temporal distribution of the relevant 221 tweets. Since this distribution is not uniform, we aim to estimate the temporal density f(T), where *T* is the vector of timestamps $t_{1},t_{2},\ldots ,t_{n}$ of the tweets. To estimate f(T), we use the Kernel Density Estimation (KDE) method [51], which is a non-parametric technique for approximating a density by analyzing data generated from that density. Given a sample $\left(x_{1},x_{2},\ldots ,x_{n}\right)$ drawn from some distribution with an unknown density *f*, the idea is to place a kernel function *K* on each data point $x_{i}$ and let the probability density function be given by the sum of *N* kernel functions. The main objective is to estimate the probability density function f(x). A histogram is a simple method to estimate the density function, but arbitrary placement of histogram bins can cause issues. Therefore, we use a kernel function *K* centred on each data point $x_{i}$ and average these functions to obtain a probability density function. This leads to a simple kernel density estimator:

(3) $$f\left(x\right)=\frac{1}{N}\sum_{i=1}^{N}K\left(x-x_{i}\right)$$

where *K* is a box function. Since each function *K* has ∫Kdx=1, we divide by *h* to ensure that ∫f(x)dx=1. Finally, the kernel density estimator is:

(4) $$f\left(x\right)=\frac{1}{Nh}\sum_{i=0}^{N}K\left(\frac{x-x_{i}}{h}\right)$$

where *K* is a symmetric, but not necessarily positive function that integrates to 1 and h>0 is the bandwidth. Though many kernel *K* functions are viable, we use the common Gaussian distribution, such that:

(5) $$K\left(\frac{x-y}{h}\right)=N\left(\frac{x-y}{h},0,h\right)$$

where *N* is the normal Density. The Gaussian was chosen for two reasons. First, it gives a ready plug-in value for the optimal bandwidth *h* and, secondly, we found through experiments that the choice of kernels has almost no effect on the performance of our methods. However, the choice of bandwidth is important, as can be noticed in Figure 3, and we rely on Sheather and Jones [52].

The figure in Figure 3 displays various kernel density estimates for the MediaEval dataset, where the yellow dots indicate relevant tweets and the black dots indicate non-relevant tweets. The Sheather and Jones bandwidth selection method is used to obtain the kernel density estimate, which minimizes the density of non-relevant tweets and reduces noise. Thus, the feature extracted in this method is represented as $F_{i}=f\left(u_{i}\right)$, where $u_{i}$ is the timestamp of a tweet, *f* is a Gaussian kernel density estimation function, and *F* is a one-dimensional feature representing the density of the corresponding tweet.

### 3.2. Sequence-Based Classification

The NGM (Neural Guided Module) is a component of the overall architecture that allows for the incorporation of external features into the sequence-based classifier (SBC) in order to regularize the loss. The SBC is a combination of a CNN and a BiLSTM, designed to capture complex associations between adjacent words and increase classification accuracy. However, incorporating external features such as BERT or VGG can further improve the performance of the model. The NGM allows for the fusion of these external features with the SBC, allowing the model to take advantage of both sequence-based and external feature-based information. The specific details of how the NGM works to incorporate these external features are described in the next subsection.

### 3.3. Neural Structure Learning

Most recent approaches suggest early or late fusion of the aforementioned representations (Equations (1) and (2) in Section 3.1 and Equation (6)) in a single layer before an additional hidden layer that lies right before the softmax layer, which computes the probability distribution over the labels. However, in the frame of the Flood-related Multimedia Task, which will be described in the next section, it was shown that such approaches are negatively affected by additional features. To tackle this issue, we introduce a novel fusion approach, that is based on a Graph Neural Network (GNN).

Its novelty can be simply explained with the following example. Note that given two different tweets, i.e., “Possible floods in Rome” and “Don’t get one of those cheap phones that seem to be flooding the market these days”, if they are not strongly connected in a graph and the first one is labelled as relevant, then the predicted probability of the second being relevant is low. In contrast, the neural network training takes into account only the labelled instances and ensures correct predictions on the training set. Thus, we use the state-of-the-art Neural Graph Machines (NGM) (Figure 2), which train a neural network architecture (SBC) by using both labelled (ground truth) and unlabelled data points (representations produced by semi-supervised techniques).

Graph-based semi-supervised learning takes as input a graph, denoted by G=(V,E,W) where *V* is the set of nodes, *E* the set of edges and *W* the edge weight matrix. Given the training label distribution for seed nodes, *Y*, the aim is to predict the labels for each node in the graph, $\hat{Y}$. For *L* labels and subject $\sum_{l=1}^{L}{\hat{Y}}_{\upsilon l}=1$ the label propagation here performs minimisation of the following:

(6) $$\begin{array}{c} C_{LP}\left(\hat{Y}\right)=\mu_{1}\sum_{u\in V_{l}}{\left|\right|{\hat{Y}}_{u}-Y_{u}\left|\right|}_{2}^{2}\\ +\mu_{2}\sum_{u\in V,u\in N(u)}{w_{u,\upsilon }\left|\right|{\hat{Y}}_{u}-{\hat{Y}}_{u}\left|\right|}_{2}^{2}\\ \mu_{3}\sum_{u\in V_{u}}{\left|\right|{\hat{Y}}_{u}-U\left|\right|}_{2}^{2} \end{array}$$

where $V_{l}$,$V_{u}$ the labelled and unlabelled nodes in graph and N(u) the neighbour node. The node *u*/*U* is the prior distribution over all labels, while $w_{u,\upsilon }$ is the edge weight between nodes *u*/*U*, and $\mu_{1}$, $\mu_{2}$ and $\mu_{3}$ are hyper-parameters.

For training deep neural networks with structured signals we are based on Neural Graph Learning [9]. The graphs were constructed using unimodal and multimodal relations from the features extracted (as described above in Section 3.1). The structured signals were used to regularise the training of the SBC for learning accurate predictions, by minimising supervised loss and the neighbour loss (Figure 2) for maintaining the similarity among inputs from the same structure.

The proposed function of NGM is a weighted sum of the neural network cost ($C_{SBC}$) produced by the text classifier SBC and the label propagation cost:

(7) $$\begin{array}{c} C_{NM}\left(\theta \right)=a_{1}\sum_{u,\upsilon \in E_{UU}}w_{u\upsilon }d\left(h_{\theta }\left(x_{u}\right),h_{\theta }\left(x_{\upsilon }\right)\right)\\ +a_{2}\sum_{u,\upsilon \in E_{LU}}w_{u\upsilon }d\left(h_{\theta }\left(x_{u}\right),h_{\theta }\left(x_{\upsilon }\right)\right)\\ +a_{3}\sum_{u,\upsilon \in E_{LL}}w_{u\upsilon }d\left(h_{\theta }\left(x_{u}\right),h_{\theta }\left(x_{\upsilon }\right)\right)\\ +C_{SBC}\left(\theta \right) \end{array}$$

where $E_{LL},E_{LU},E_{UU}$ are sets of labelled-labelled, labelled-unlabelled and unlabelled-unlabelled edges correspondingly, $h_{\theta }$ represents the hidden representations of the inputs produced by the neural network and d() is $l_{2}$ distance metric and $a_{1},a_{2},a_{3}$ are hyper-parameters.

## 4. The Flood-Related Multimedia Dataset & Task

In this section we present the preparation of a new dataset that comprises Italian tweets, manually annotated as relevant or not to flood disasters, and we highlight its contribution. We also describe the organisation of a related evaluation task that involved this particular dataset and the lessons learnt from the results of the participating teams.

### 4.1. Dataset Preparation

The creation of the dataset that is used in our experiments (Section 5) constitutes also part of our work. The dataset consists of 7698 social media posts that have been collected from the Twitter platform between the years 2017 and 2019, by searching for Italian keywords inside their text that are related to flooding incidents (complete list in Table 2), without any other filtering operations. The keywords were proposed by employees of the Eastern Alps River Basin District (http://www.alpiorientali.it/ (accessed on 1 April 2023)), who are experts on flood risk management in the Eastern Alps district of Northeastern (NE) Italy. The motivation behind this selection is the frequent flood events in cities of NE Italy, such as Venice, Vicenza, and Trieste, as well as surrounding areas. In addition, the involvement of the Italian language aims to encourage researchers to move away from a focus on English.

The ground truth data of the dataset concerns the relevance of each tweet to an actual flood, since the keyword-based search can result in posts that contain flood-related keywords, but in reality, their content is irrelevant, e.g., in the case that a term is used metaphorically. Examples of relevant and non-relevant tweets can be seen in Figure 1. Human annotation was performed to label tweets as relevant (1) or not relevant (0), giving greater importance to text over image. The annotators were again the employees of the Eastern Alps River Basin District, whose expertise permitted them to mark as relevant tweets that mentioned specific events in their district, known a priori to them as real; moreover, the text of the tweets was in their native language, i.e., Italian. It should be noted here that each tweet has been annotated by a single individual, while in the cases where one of the modalities of the tweet is relevant and another is not (e.g., a tweet with relevant text and irrelevant image and vice versa) the annotators have given more weight to the text.

**Figure 4:**
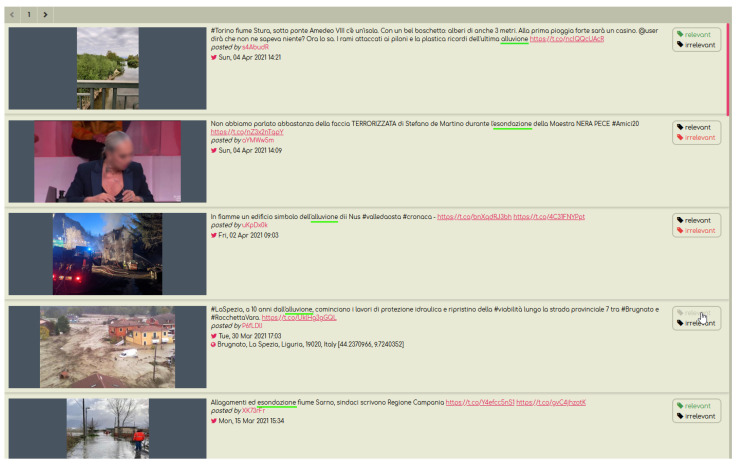
Screenshot of the annotation tool.

To assist the experts with the annotation process, we have developed a dedicated tool as a Web application (Figure 4). The application provided a straightforward way to label posts, by simply clicking the “relevant” or “irrelevant” buttons next to each tweet. The final outcome, after using the annotation tool, was 21% of the tweets marked as relevant and 79% marked as not relevant (for details see Table 3).

The key points of the dataset’s contribution are listed here:

- All tweets contain both textual (Twitter message) and visual information (attached image), which makes the dataset fitting for evaluating fusion techniques.
- The dataset is dedicated to the natural disaster of floods and expands to posts published from 2017 to 2019.
- The tweets are manually annotated as relevant/non-relevant by experts, thus they can be used as a high-quality training dataset for relevance classification.
- The tweets are in Italian, so the dataset overcomes the usual limitation of training data being in English.

### 4.2. Dataset Release

The described dataset has been released in the frame of the MediaEval Multimedia Evaluation benchmark 2020 (https://multimediaeval.github.io/editions/2020/ (accessed on 1 April 2023)) and, in particular, the Flood-related Multimedia Task (https://multimediaeval.github.io/editions/2020/tasks/floodmultimedia/ (accessed on 1 April 2023)). The objective of this task was to build a binary classifier that is able to distinguish whether a tweet is relevant or not to a flooding event in the examined area. Participants were allowed, through five separate submission runs, to tackle the task by using text features, image features, as well as a combination of both.

Upon release, the dataset was divided randomly into two subsets (Table 3): *development-set*, which included 5419 tweets and was provided to participants together with ground truth labels for training purposes, and *test-set*, which comprised 2279 tweets without their annotation for evaluation. Both subsets along with the complete ground truth (labels for test-set were provided after the completion of MediaEval 2020) are available on the task’s GitHub repository (https://github.com/multimediaeval/2020-Flood-Related-Multimedia-Task (accessed on 1 April 2023)). In order to be fully compliant with the Twitter Developer Policy (https://developer.twitter.com/en/developer-terms/agreement-and-policy (accessed on 1 April 2023)) that disallows sharing collected data with third parties, solely the IDs of the tweets are distributed and those interested can use them to retrieve the posts and their associated images. For more information on the Flood-related Multimedia Task, the reader is referred to [15].

### 4.3. Lessons Learnt

Conducting an evaluation task has yielded significant knowledge about the classification problem of relevant/non-relevant posts. First of all, the proposed solutions of the five participating teams, i.e., FIG [53], UEHB-ML [54], QUT [55], fastNuDs [56], and HBKU_UNITN_SIMULA [57] (all described in Section 5), showed that there are several alternative approaches regarding text representation (e.g., BOW, TF-IDF, BERT), classification (e.g., MLP, RNN, CNN, SVM, RF, MNB), and over/under-sampling (e.g., SMOTE, Augmentor, random). Based on the scores of all teams, it has been proven to be a very hard task, while the subjectivity of the annotators strongly affected the results. Nevertheless, focusing on the textual features generally led to better performance, especially when the solution considered undersampling.

## 5. Experiments

In this section we present the considered baselines, along with their best runs, the settings of our model and, finally, the results of the comparison between our approach and the baselines.

### 5.1. Baselines

Our model is compared against baseline text classification methods (BERT, CNN, BiLSTM) and in multimodal approaches which proved great results in similar text classification tasks that was reproduced for evaluation in the corresponding dataset:

- BERT [31] is based on transformer encoder architectures and has been successfully used on a variety of tasks in NLP (natural language processing). They compute vector-space representations of natural language that are suitable for use in deep learning models. BERT models are usually pre-trained on a large corpus of text, then fine-tuned for specific tasks.
- CNN [58] classify text by working through the 1-dimensional convolving filters which are used as ngram detectors and each filter specializing in a closely-related family of ngrams. Afterwards, max-pooling over time extracts the relevant ngrams for making a decision. The rest of the network classifies the text based on this information.
- BiLSTM proved to be effective with high accuracy in text classification tasks [59] compared to RNN, CNN and LSTM.
- CNN+BiLSTM [44] propose an hybrid model that capitalize on the advantages of LSTM and CNN for text classification.
- CNN+VGG16 [34] propose to use both text and image modalities of social media data to learn a joint representation using state-of-the-art deep learning techniques.

Additionally, we compare the methodologies that were implemented and evaluated in the context of the Flood-related Multimedia Task. These particular solutions have been selected as they all constitute very recent works and they can be easily compared on the dataset of the contest, i.e., the one presented in this paper (Section 4.1). A short description of the these baselines follows:

- FIG [53] undersampled the dataset in order to have a more balanced set. Additionally, text features were extracted with CNN and LSTM models and then combined by Multi-Layer Perceptron before the single output unit. Contrary to that, to address the individual weaknesses and leverage the distinct advantages of LSTM and CNN, we used the SBC hybrid RNN model.
- UEHB-ML [54] used Synthetic Minority Oversampling Technique (SMOTE) [60] for the imbalanced problem. In feature extraction, they used pre-trained CNNs for the images and Bag-of-Words for text. Finally, they combined the visual and textual information in a late fusion scheme. Alternatively, we used knowledge graphs whose nodes represent the particular features (i.e., text, image, time).
- QUT [55] early fused the textual and visual features extracted from Bag-of-Words and pre-trained Xception on the ImageNet dataset. We employ BERT and VGG19 for feature extraction.
- Fast-NuDs [56] used Augmentor [61] for the imbalanced dataset, which creates multiple copies of each image. For the training, VGG16 has utilised hybrid weights of Places365 [62] and ImageNet for the visual features and TF-IDF with Multinomial Naïve Bayes for the textual features. Finally, the average of each outcome was used for late fusion. Opposed to that, we used undersampling and focused on textual features, which generally led to better performance.
- HBKU_UNITN_SIMULA [57] used BERT in the text network and ResNet152 for the image network. In parallel, object and scene-level features were extracted through VGGNet16 and were pre-trained on ImageNet and Places Datasets. All these features were early fused during the training. In our model, we consider temporal features as external information to enhance its performance.

### 5.2. Settings

The overall hyperparameters and constants of the architecture are provided in Table 4. In fact, the task concerns two classes (0 for non-relevant, 1 for relevant). Since this is a binary classification problem and the model outputs a probability (a single-unit layer with a sigmoid activation), we’ll use the binary crossentropy loss function with adam optimizer. Regarding the SBC model, in Table 4 listed the corresponding CNN filters and kernel size as well as the BiLSTM settings.

On the other hand, the sparse graphs, for the NGM input, were constructed with one or more different embeddings and the similarity between these embeddings was computed to generate a response message graph with a fixed node degree (k = 10). Particularly, the resulting data include original sample features (image feature, text feature, KDE feature) as well as features of their corresponding neighbours. We consider undirected edges and use a maximum of 3 neighbours per sample to augment training data with graph neighbours as well as the graph regularization multiplier equal to 0.001 which is the relative weight of the graph regularization term in the overall loss function. For the graph construction, we use the graph builder’s support for locality-sensitive hashing (LSH) (https://www.tensorflow.org/neural_structured_learning/api_docs/python/nsl/tools/build_graph_from_config (accessed on 1 April 2023)) to speed up the process. Every abstract representation of the embeddings corresponds to a unique node and the weighted edges to similarity score. The similarity score is computed with the Euclidean distance (L2). Regarding the Word Embedding layer, we use pre-trained embeddings for Italian language (https://wikipedia2vec.github.io/wikipedia2vec/pretrained/ (accessed on 1 April 2023)). As our corpus contains words that are missing from the pre-trained model, these lacking words are randomised with uniform (−0.25, −0.25, dim), where dim is the dimension of the word vector (all sentences padded to 50). The vocabulary size of our dataset is 14,313 unique words, with around 2514 words not found in the Word2Vec word embeddings. In order to capture the text representation of the whole tweet, we use an existing pre-trained model in the Italian language called ‘Bert-base-italian-xxl-cased’ (https://huggingface.co/dbmdz/bert-base-italian-cased (accessed on 1 April 2023)). Thus, the input in the BERT model is a Twitter text and the output is a feature vector with dimension H=1×768. Additionally, we use VGG19 convolutional neural network model, pre-trained on ImageNet to process images with dimension D=1×512.

**Figure 5:**
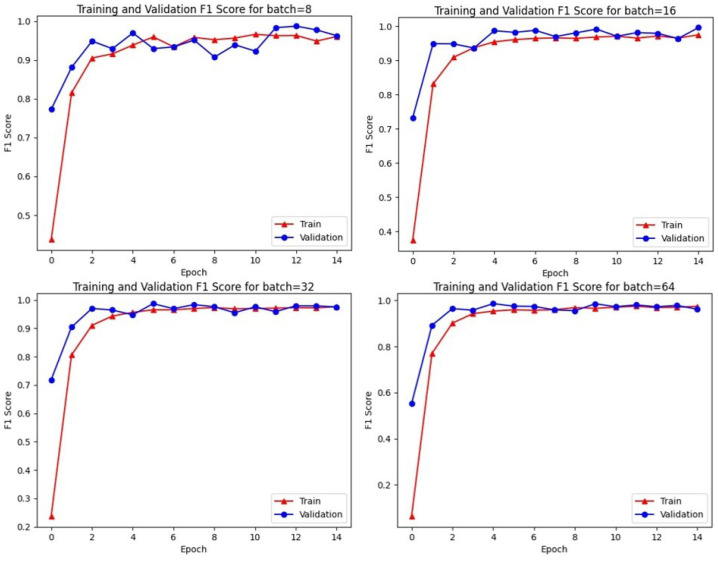
Model learning rate performance on different batch sizes.

As the dataset is imbalanced (positive 21%, negative 79%), it can affect negatively our model and lead to poor performance by ignoring the minority class. To tackle this issue, random under-sampling has shown great results [63] by selecting and removing randomly some of the negative samples, in order to have the most balanced possible dataset with a class ratio closer to one. The ratio is a hyper-parameter that has been fine-tuned to 0.7. Early stopping was used for minimum f1 in three patience steps in 15 epochs. We evaluate the performance using the well-known F1 score in classification problems. Finally, cross-validation was used for evaluating the performance of the classifier. The general procedure begins by splitting the training dataset into random sets (train, validation and test). Particularly, we randomly split the dataset to 90% for the train set and 10% for the test set and the validation set are randomly split to the 10% of the training set. Finally, the batch size was selected to 64 due to the learning rate performance of the model as Figure 5 presents.

### 5.3. Results

In the Flood-related Multimedia Task participants were allowed to submit up to five runs, with the first focused only on textual features, the second on text-image fusion, and three open runs, which also allowed the usage of external features. However, there was not a proposed solution that involved additional features, apart from text and images. In fact, the task proved to be hard for the participants (max F-score = 0.5405, avg = 0.2183) and the best performance was achieved by using textual features only (FIG [53]).

Table 5 presents the results of participants’ methods within the Flood-Related Multimedia Task, also in comparison with our work. Besides the proposed work (SBC + NGM), we implemented an additional method that early fuses the external features with the sequence-based features extracted from SBC in a shared representation before the classification. Both the proposed and the additional method are mentioned with “(OURS)” in the table. Moreover, the features fused with the NGM method are presented as “NGM_feature_”. The results are divided into three parts: text-only approaches in the first part, text and image in the second, and text with external features in the last one. Starting with the text-only part, FIG [53] is the best performing approach among the participants. As mentioned before, FIG early fused the features extracted from CNN and LSTM. In comparison to our SBC hybrid model, we observe that our implementations are slightly outperforming FIG’s approach, with our idea to fuse BERT features in the Sequence-Based model proved to be the most efficient (SBC + NGM(BERT), F1 = 0.5480).

There is a general assumption that models perform better by adding more and more features. Fusing visual features, fastNuDs [56] achieved the best results. They used a late fusion method to combine visual and text features. Nevertheless, the results in this particular dataset show that fusion with different modalities does not offer a lot, apparently due to the noise of the external features (e.g., images). For instance, many tweets are labelled as relevant based on their text, but their attached image is not relevant, affecting in this way the validation part. However, our proposed approach which fuses the features with the NGM method (SBC + NGM_VGG19+BERT_, F1 = 0.5158) significantly outperforms in comparison with early/late fusion methods, showing that the irrelevant visual features do not heavily affect the model’s performance.

In the specific task that we have focused on, our GNN method has shown superior performance compared to existing approaches. Our GNN method introduces a novel way of incorporating graph structure information into the learning process. Unlike previous methods that rely on traditional convolutional operations, our method leverages the attention mechanism to dynamically weigh the importance of different nodes and edges in the graph. This approach allows our model to better capture the complex relationships between nodes and their neighborhoods, resulting in more accurate predictions. Furthermore, our GNN method utilizes a multi-head attention mechanism, which allows it to learn multiple representations of the graph at different levels of abstraction. This enables the model to capture both local and global patterns in the graph, resulting in a more comprehensive understanding of the data. Finally, we have shown through extensive experiments on benchmark datasets that our GNN method outperforms state-of-the-art approaches in terms of accuracy and efficiency. These results clearly demonstrate the novelty and effectiveness of our proposed GNN method for the task at hand.

## 6. Discussion

Besides the visual features, we also examined an external feature extracted from Kernel Density Estimation (KDE). As described in Section 3.1, KDE takes as input the publication date/time of the tweet (timestamp) and extracts a single value feature. According to the results, KDE proved to be a valuable feature by comparing the (SBC + NGM_VGG19+BERT_, F1 = 0.5158) with (SBC + NGM_BERT+VGG19+KDE_, F1 = 0.5379) and taking into account the temporal dynamics that a crisis event involves and the variety of concepts that may be depicted in a relevant tweet. In particular, the NGM fusion with text, image and KDE (SBC + NGM_BERT+VGG19+KDE_, F1 = 0.5379) outperforms all the multi-modal fusion methods.

Recently the utilisation of graph neural networks has shown promising results in the field of social media classification [64]. Thus, more benchmark datasets and novel approaches can lead to the expansion of the knowledge around multimodal fusion challenges. In this context, the organisation of the Flood-related Multimedia Task provided the research community with a novel challenge for multimedia classification as well as a benchmark dataset, which consists of Italian tweets annotated by experts with regards to their relevance to floods, to be generally used for training and evaluation. Furthermore, the results of the participating teams offered us important insights into the multimodal classification problem. In this work, we also investigated and implemented a novel method using graphs that can utilise multimodal information to disambiguate the mentions in a fine-grained way, mine the hidden and explicit relationships across modals, and reduce the noise of each modal. Our proposed approach outperforms all recent works in the three different challenges of the task.

Future work will concentrate on novel strategies for fusing features with graph neural networks and the involvement of alternative features (e.g., weather data, geographic information). Another direction will be the extension of this work to cover the relevance estimation of Twitter posts that relate to other natural disasters, such as fires and earthquakes.

## Figures and Tables

**Table 1:** Summary of related works in multimodal fusion.

Research	Data Type	ML Algorithms	Multimodal Fusion
de Bruijn et al. (2020) [27]	Text, Hydro-logical	fastText [28], MUSE [29]	Late Fusion
Mouzannar et al. (2018) [30]	Text, Image, Video	Inception, CNN	Late Fusion
Gautam et al. (2019) [32]	Text, Image	BiLSTM, Inception-v3, Logistic Regression Decision	Late Fusion
F. Ofli, et al. (2020) [34]	Text, Image	VGG16, CNN	Early Fusion
M. Abavisani, et al. (2020) [39]	Text, Image	DenseNet [40], BERT [31]	SSE-GRAPH [43]
The proposed method	Text, Image, Time	BERT [31], CNN, BiLSTM, VGG19	NGM [42]

**Table 2:** Keywords to retrieve tweets.

Italian Keywords	English Translation
allagamento	flooding
allerta meteo	weather alert
allerta meteo vicenza	weather alert Vicenza
alluvione	flood
alluvione vicenza	flood Vicenza
bacchiglione	Bacchiglione (river)
esondazione	flood
fiume piena	full river
livello fiume	river level
sottopasso allagato	flooded underpass

**Table 3:** Number of annotated tweets per set.

Set	Relevant	Not Relevant	Total
Development-set	1140 (21%)	4279 (79%)	5419
Test-set	492 (22%)	1787 (78%)	2279
Complete set	1632 (21%)	6066 (79%)	7698

**Table 4:** Hyperparameters and constants used for training and evaluation.

Hyperparameter	Value
classes	2
max sequence length	50
distance type	L2
graph regularization multiplier	$1\times 10^{-3}$
neighbors	3
CNN filters	100
CNN kernel size	5
CNN activation	tanh
CNN kernel regularizer	$L2\;(1\times 10^{-3})$
BiLSTM units	64
BiLSTM droput	0.2
Optimizer	adam
loss	binarycrossentropy
train epochs	15
batch size	64
callback	early stop

**Table 5:** Experimental comparison.

Method	Features	Fusion	F1
FIG [53]	Text	-	0.5405
UEHB-ML [54]	Text	-	0.4376
UT [55]	Text	-	0.4158
fastNuDs [56]	Text	-	0.3631
HBKU_UNITN_SIMULA [57]	Text	-	0.2120
CNN+BiLSTM [44]	Text	-	0.5425
CNN	Text	-	0.2178
BiLSTM	Text	-	0.2002
BERT [31]	Text	-	0.1514
(OURS) SBC + NGM_BERT_	Text	NGM	**0.5480**
fastNuDs [56]	Text + Image	Late Fusion	0.2786
QUT [55]	Text + Image	Early Fusion	0.1478
UEHB-ML [54]	Text + Image	Late Fusion	0.0936
HBKU_UNITN_SIMULA [57]	Text + Image	Early Fusion	0.0462
CNN+VGG16 [34]	Text + Image	Early Fusion	0.2442
CNN+BiLSTM+VGG19	Text + Image	Early Fusion	0.2267
(OURS) SBC + NGM_VGG19+BERT_	Text + Image	NGM	**0.5158**
(OURS) SBC	Text + Time	Early Fusion	0.4932
(OURS) SBC	Text + Image + Time	Early Fusion	0.1735
(OURS) SBC + NGM_VGG19+KDE_	Text + Image + Time	NGM	0.5270
OURS) SBC + NGM_BERT+VGG19+KDE_	Text + Image + Time	NGM	**0.5379**

## Data Availability

The reported data can be found at https://github.com/multimediaeval/2020-Flood-Related-Multimedia-Task (accessed on 1 April 2023).

## Abbreviations

The following abbreviations are used in this manuscript:

KDE	Kernel Density Estimation
NGM	Neural Graph Machines
SBC	Sequence-Based Classification
GNN	Graph Neural Network
